# Health improvement and prevention study (HIPS) - evaluation of an intervention to prevent vascular disease in general practice

**DOI:** 10.1186/1471-2296-11-57

**Published:** 2010-08-05

**Authors:** Mahnaz Fanaian, Rachel A Laws, Megan Passey, Suzanne McKenzie, Qing Wan, Gawaine Powell Davies, David Lyle, Mark F Harris

**Affiliations:** 1 Centre for Primary Health Care and Equity, School of Public Health and Community Medicine, University of New South Wales, Sydney NSW 2052, Australia

## Abstract

**Background:**

The Health Improvement and Prevention Study (HIPS) study aims to evaluate the capacity of general practice to identify patients at high risk for developing vascular disease and to reduce their risk of vascular disease and diabetes through behavioural interventions delivered in general practice and by the local primary care organization.

**Methods/Design:**

HIPS is a stratified randomized controlled trial involving 30 general practices in NSW, Australia. Practices are randomly allocated to an 'intervention' or 'control' group. General practitioners (GPs) and practice nurses (PNs) are offered training in lifestyle counselling and motivational interviewing as well as practice visits and patient educational resources. Patients enrolled in the trial present for a health check in which the GP and PN provide brief lifestyle counselling based on the 5As model (ask, assess, advise, assist, and arrange) and refer high risk patients to a diet education and physical activity program. The program consists of two individual visits with a dietician or exercise physiologist and four group sessions, after which patients are followed up by the GP or PN. In each practice 160 eligible patients aged between 40 and 64 years are invited to participate in the study, with the expectation that 40 will be eligible and willing to participate. Evaluation data collection consists of (1) a practice questionnaire, (2) GP and PN questionnaires to assess preventive care attitudes and practices, (3) patient questionnaire to assess self-reported lifestyle behaviours and readiness to change, (4) physical assessment including weight, height, body mass index (BMI), waist circumference and blood pressure, (5) a fasting blood test for glucose and lipids, (6) a clinical record audit, and (7) qualitative data collection. All measures are collected at baseline and 12 months except the patient questionnaire which is also collected at 6 months. Study outcomes before and after the intervention is compared between intervention and control groups after adjusting for baseline differences and clustering at the level of the practice.

**Discussion:**

This study will provide evidence of the effectiveness of a primary care intervention to reduce the risk of cardiovascular disease and diabetes in general practice patients. It will inform current policies and programs designed to prevent these conditions in Australian primary health care.

**Trial Registration:**

ACTRN12607000423415

## Background

The prevention and treatment of cardiovascular disease are major challenges confronting the community and the health system in Australia and internationally [1]. Given the contribution of cardiovascular disease to disease burden, the cost of inadequate prevention is high [2]. Diabetes, itself a major risk factor for ischaemic heart disease and stroke, affects 7.5% of Australians, and a further 16.3% of the population over the age of 25 years have impaired glucose metabolism [3].

There is considerable evidence for the effectiveness of interventions to prevent cardiovascular disease at the population and individual levels [4]. A simple office-based lifestyle intervention has been shown to significantly reduce total cholesterol in hypercholestrolaemic patients [5]. Intensive lifestyle interventions involving diet and exercise have also been shown to be effective in the prevention of diabetes in patients with impaired glucose tolerance and also lowering blood pressure in patients with hypertension over 6 months [5-8]. Even moderate reductions in weight has been shown to reduce the incidence of diabetes by more than one half [7-9]. Studies such as these suggest that there is a strong case for intervening in groups at high risk of developing lifestylerelated disease [10].

The Australian national health survey 2007-2008 found that the most common vascular risk factors were those related to nutrition, with over 90% of adults not meeting the recommended serves of vegetables, over half not consuming adequate amounts of fruit and 62% being overweight or obese [11]. Around one third of adults were classified as physically inactive, one in five smoked, and of the 59% of the population who drank alcohol, 21% did so at a level which would pose a risk to their health [11]. Patients who already have one or more risk factors for vascular disease may be more likely to perceive potential health risk, as well as benefits, than those who are not at risk. This may increase their motivation to participate in lifestyle change activities [12]. However, advice about risk may also increase psychological distress [13] which in turn can also impact on behaviour choices [14].

General practice is well placed to offer early interventions to modify lifestyle risk factors. It provides care across the continuum from prevention of illness to treatment and rehabilitation, and provides consultations to approximately 90% of Australians each year [15]. In 2007-08, 59% of general practice encounters were with patients who were overweight or obese, 26% with those who drank alcohol at risky levels and 17% with those who smoked daily [16]. There is evidence that lifestyle modification can be implemented in general practice and that selective interventions can bring about behaviour change in patients [17-19]. However, many studies have focused on single risk factors and have not tried to change systematically. Findings from research into interventions that have targeted multiple factors have been more equivocal [20].

Few primary care encounters in Australia involve risk-factor assessment and intervention, signifying an important gap between evidence and practice [21]. The challenge is thus to determine if interventions outside the practice can be integrated with routine clinical care in Australian general practice. Our previous research and that of others suggests that referral of high risk patients to services to support lifestyle change is infrequent [22-24].

General practice preventive care in Australia has recently been enhanced by the introduction of health checks. A "Well Person's Health Check" in general practice for people aged 45-49 was introduced in November 2006, as a once-only service for those who have one or more identifiable risk factors for chronic disease [25]. In 2008 a diabetes risk check was introduced for patients aged 40-49 which aimed to identify patients at high risk for diabetes, to introduce lifestyle interventions. Although these checks have been taken up by GPs, the referral rate of high risk patients for more intensive lifestyle interventions has been disappointingly low [26].

### Study Aim and Hypotheses

The aim of the HIPS is to evaluate the impact of a general practice intervention for patients at high risk of vascular disease on changes in behavioural and physiological risk factors (Table 1).

**Table 1:** Study hypotheses

Primary Hypotheses	Secondary Hypotheses
1. In the intervention group 20% more patients who are at high risk of developing vascular disease will be offered evidence-based interventions (motivational counselling or referral to appropriate services) to modify their risk factors compared with patients in the control group.	4 Patients in the intervention group will be more likely to progress in their stage of change compared with the control group.
2 In the intervention group the self-reported attitudes and practices of general practice staff towards preventive care and behavioural risk factor management will improve compared with the control group	5 In the intervention group, a lower proportion of patients with high psychological distress (K10 score > 15) will change their behaviour compared to those who have low psychological distress (K10 score < 15)
3 The behavioural (diet, physical activity, smoking and alcohol) and physiological (weight, LDL cholesterol, blood pressure) risk factor scores of patients identified at high risk in the intervention group will be reduced over 12 months in comparison with high risk patients in the control group.	

## Methods/Design

The HIPS is a cluster-randomised controlled trial with randomisation at the practice level, conducted in general practice in the state of New South Wales (NSW), Australia.

### Recruitment

#### Recruitment of Divisions of General Practice and Practices

**Figure 1:**
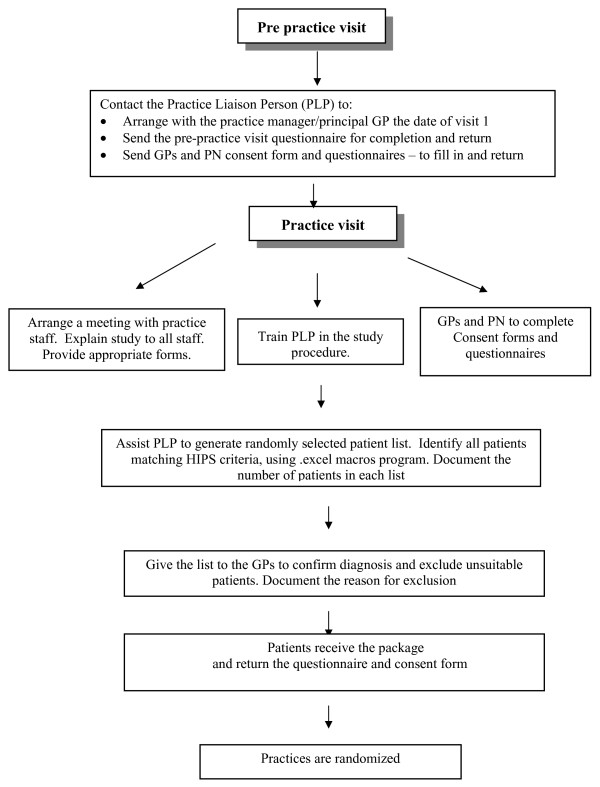
**HIPS Recruitment Flowchart**.

In Australia, general practice is supported by regional organizations known as Divisions General Practice (DGPs), part of whose role is to work with practices to improve their quality of care. This study involves three urban and two rural DGPs in NSW, which invited practices to participate in the study. Practices are identified from databases held at the DGP and are invited to participate in the study via a letter from the Division. Each DGP is asked to recruit eight practices to participate in the study (two of the urban DGPs participate as a pair, each aiming to recruit four practices). General practices are eligible to participate if they use computer-based medical records, are not involved in other research, and are located in the participating DGPs. All GPs and PNs in participating practices are invited to take part in the study (Figure 1).

#### Recruitment of Patients

Participating practices are asked to search their electronic records to identify a list of 160 patients who meet the selection criteria (Table 2). Previous research suggests that 25% of these will be eligible and willing to participate. The rationale for the eligibility criteria is to recruit sufficient patients with high to moderate risk of cardiovascular disease or diabetes to participate in the full intervention (both in general practice and the referral program). Patients in the 40-55 age groups are targeted by existing health checks but are less likely to have multiple risk factors or to be at high CVD or diabetes risk. Thus in this age group we are specifically targeting those who already have physiological risk factors. Patients aged 56-64 are more likely to have multiple risk factors and also to be at increased risk because of their age. The majority of general practice patients older than 64 years have already developed a chronic disease.

**Table 2:** Patient selection criteria

Inclusion criteria	
• Aged 40-55 with hypertension and/or hyperlipidaemia	
• Aged 56-64	
• Attended the practice in the last 12 months	

**Exclusion criteria by practice**	**Exclusion criteria by GP**

Diagnosed or treated for:	• Has current severe illness or personal circumstances which are of overriding concern;
• Diabetes	
• Cardiovascular disease	• Is deceased (up to date medical records generally prevent this)
• Renal disease	• Is no longer a patient of the practice;
• Stroke	• Is unable to speak adequate English;
	• Has diabetes or cardiovascular disease;
	• Is unlikely to be able to read and understand the information sheet and consent form because of significant cognitive impairment (e.g. dementia.)

Up to 160 eligible patients are sent a letter from the practice, signed by their GP, advising that the practice is participating in the research and that the patient has been randomly selected to be invited to participate (if the list exceeds 160 patients, 160 are randomly selected for recruitment). An information sheet, consent form, questionnaires and a stamped envelope addressed to the project coordinator are also included. Patients are asked to read the information, sign the consent forms, complete the questionnaires, and return them if they wish to take part (Figure 1). When patients consent to participate, a letter of invitation to attend their GP for a health check and blood test form is mailed to them. The cost of the blood test and the GP visit is reimbursed by the study.

### Randomisation and Blinding

After baseline data collection, the 30 practices are randomly allocated in variable blocks (stratified by DGP) to 'intervention' or 'control' groups by a person independent of the research team using computer generated random numbers. Staff involved in the data collection are independent of those involved in the intervention and are not informed which practices are randomized to intervention or control groups.

### Intervention

The main aim of the intervention is to assist high risk patients to make positive lifestyle changes through supporting self-management knowledge and skills, providing social support and increasing self-efficacy. At the practice level, this includes training practice staff in assessing risk factors and motivational interviewing using simulated consultations with actors and practice support. Patient education resources include resources from the Lifescripts program [27], patient waiting room questionnaires, a Health check visit guide, a checklist for GPs to complete when they see patients, and a food diary (patients are asked to fill out 3-4 days of their diary intake each week, including at least one weekend day, in the Daily Living Diary provided).

At the patient level, the goals of intervention are based on those used in previous diabetes prevention studies, including the U.S. Diabetes Prevention Program (DPP) and the Finnish Diabetes Prevention Study (DPS) [6,7,9] (Table 3).

**Table 3:** HIPS intervention goals

	Intervention Goals
Exercise	• Moderate exercise for at least **30 minutes/day**, including walking, jogging, swimming, aerobics, ball games, or skiing with circuit-type resistance training, twice a week.

Diet	• Diet low in saturated fats, sucrose and salt with increased portions of vegetables and fruit per day (up to 7 portions) in order to achieve a diet with the percentage of energy from carbohydrate = 50%, saturated fat < 10% and total fat < 30%, protein 1 g/kg ideal body weight per day, fibre 15 g/1000 kcal.

Weight reduction	• (if overweight) of ≥ 5 kg or 5% of body weight

Smoking cessation	• if smoker

Limit alcohol intake	• (if drinking) to ≤ 2 drinks/day, including 1-2 alcohol free days/Week

#### GP/PN brief intervention

This intervention is modelled on previous research conducted by the investigators, called SNAP (Smoking, Nutrition, Alcohol, Physical activity) interventions in general practice [22], and evaluation of the 45-49 year health check [28]. The aim is to develop a brief intervention that can be carried out by busy GPs and their practice staff at the time of a "health check" such as that currently supported for patients aged 45-49 years [29] (Table 4). The key theoretical underpinning for the GP intervention is the Trans-theoretical (Stages of Change) Model which focuses on the assessment of patient readiness to change and stage-based tailoring of brief behavioural counselling [30]. This is applied within the framework of the 5As model [31] (Table 5). A broad definition of high risk (Table 6) is used (presence of a physiological risk factor, overweight or smoking).

**Table 4:** HIPS Health Check Visit

Health Check Visit - checklist
• SNAP lifestyle risk factor assessment and assessment for previous GDM
• Review of pathology reports (lipids, fasting glucose) and/or ordering of these
• Physical assessment (weight, height, BMI, waist circumference)
• Assessment of readiness to change
• Interventions offered (education, medication change)
• Referral of high risk patients for nutrition/physical activity program
• Follow up visit with GP at about 10 - 12 weeks

**Table 5:** 5As model

Ask	Identification of eligible patients who are 40-64 years from practice records with invitation to attend the practice for a health check following a blood test for fasting glucose and lipids. Completion of a waiting room questionnaire by patients on their risk factors
**Assess**	Assessment of behavioural and physiological risk factors and readiness to change

**Advise**	Brief advice using written materials (lifescript resources)

**Assist**	Motivational counselling and medications if appropriate

**Arrange**	Referral of high risk patients who are unsure or are ready to change to an allied health provider (either an exercise physiologist or a dietician) for individual advice and goal setting; and to the *CHANGE for HIPS *group program. Prior to attending the program patients are asked to complete a food diary. Follow up is arranged with the GP at about 10 - 12 weeks.

**Table 6:** Definition of high risk patient

Definition of High Risk patient
High risk patients are defined as those with any of the following characteristics:
• have history of gestational diabetes (GDM)
• have 'Pre diabetes' - (impaired glucose tolerance or impaired fasting glycaemia)
• have elevated blood pressure (BP ≥ 140/90 on two occasions) or on treatment for high blood pressure
• have high Lipid: Total Cholesterol (TC) > 4.5 mmol/L or Low Density Lipoprotein (LDL) > 2.5 mmol/L or Triglyceride (TG) > 2.0 mmol/L or on treatment for it
• are overweight: BMI > 28 or waist circumference > 102 cm in males and 88 cm in females
• currently smoke.

#### Development of the Intervention Program for High Risk Patients

##### Individual lifestyle sessions

Patients at high risk are asked to attend an initial visit, at which an allied health practitioner (AHP) reviews their food diary and negotiates individual dietary and physical activity goals with the patient. A follow-up visit with the AHP is arranged at around 8-9 weeks to review progress and negotiate additional dietary and physical activity goals.

##### Group Lifestyle Education - CHANGE for HIPS

**Figure 2:**
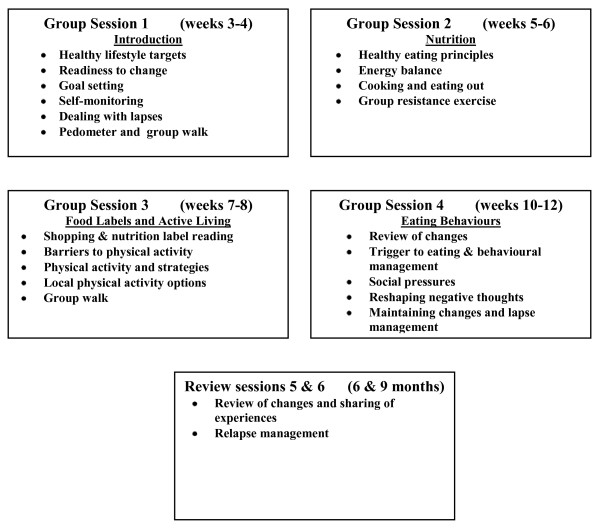
**CHANGE for HIPS**.

After attending the AHP, patients are enrolled the *CHANGE for HIPS*, This is a group education program, which is adapted from the group component of the "*Counterweight Program - CHANGE*" in the UK [32]. An intervention officer (IO) from the local DGP is trained to facilitate this program and provided with the required resources. *CHANGE for HIPS *consists of four group sessions (1.5 hours each) over the first three months and a further two follow-up sessions at six and nine months. The group sessions include an educational and physical activity component (20-30 minutes of walking or resistance exercise) and are based on the use of self-management strategies (goal setting, selfmonitoring, and developing practical skills and problem solving to promote positive dietary and physical activity changes and weight loss) (Figure 2). Between sessions patients keep a food and physical activity diary, use a pedometer and carry out home-based physical activity.

#### Quality assurance

The quality of the intervention process is monitored by an Intervention Advisory Committee It includes evaluation of the GP/PN training sessions, evaluation of group sessions in each DGP, record of practice visits kept by IOs and ongoing feedback by practice staff on the intervention, and evaluation of patients' feedback on group sessions.

#### Usual care

Patients attending the control practices receive usual general practice care of their risk factors. After the second data collection at 12 months, the HIPS GP/PN training is offered to GPs in those practices.

### Data collection

Data are collected at baseline, 6 months and 12 months from administrative staff, GPs, PNs, and patients. The questionnaires take between 10-30 minutes to complete (Table 7). These questionnaires have been piloted before their use in the trial.

**Table 7:** Study instruments

Measurement	Details	Timeframe
**Practice questionnaire (PPVQ)**	• Practice capacity for preventive care including use of patient education materials, staff roles and teamwork in preventive care, and linkages between the practice and support services	**Baseline**

**GP/PN survey**	• This survey is based on questions from the *Preventive Medicine Attitudes and Activities Questionnaire (PMAAQ) *[38], and the *Vascular Interventions Survey *[39]	**Baseline & 12 Month**

**Patient's survey**	• This survey is based on the NSW Health Survey and previous research [40,41]. It includes questions about: (1) Practice attendance; (2) reported assessment and management of SNAP risk factors; (3) attendances at other services as a result of referral from the practice or self-referral; (4) self-reported fruit and vegetable intake, smoking, physical activity and alcohol intake, and attempts to change these; (5) readiness for behaviour change (stage of change) for each SNAP risk factor [42]; (6) The Kessler Psychological Distress Scale (K-10), a ten item questionnaire measuring negative emotional states in the preceding four weeks [43]; (7) The Neuroticism Scale from the Eysenck Personality Questionnaire Revised, Short form (EPQ-N-RS) [44], which represents an individual's tendency to experience psychological distress or neuroticism; (8) satisfaction with their care, using the General Practice Assessment Questionnaire (GPAQ) which includes questions in relation to satisfaction with GP and AHP visit and health check [45]	**Baseline & 6 months & 12 month**

**Patient's physical assessment**	• Includes weight, height, BMI, waist circumference and blood pressure measurement.	

**Patient's fasting blood tests**	• Patients are asked to have a fasting blood test to assess their serum lipids (total cholesterol, HDL, LDL, triglycerides) and glucose.	**Baseline & 12 month**

**Clinical record audit**	• Patient records are examined to determine recorded weight, waist circumference, blood pressure, practice attendances during the 12 months of the trial, referral to interventions to address risk factors identified, prescribing of medications (lipid lowering and antihypertensive medications), referrals to dietician, exercise and smoking cessation program.	**12 month**

**GP/PN & IOs interviews**	• Qualitative interviews are conducted with GPs and PNs in the intervention group and with IOs to explore experience of implementing SNAP risk factor management in routine practice, feasibility and acceptability of brief lifestyle intervention, as well as acceptability and usefulness of referral to support services.	**12 month**

#### Sample size calculation

All sample size calculations use a significance level of 5% and power of 80%, with design effects and estimated expected differences between the intervention and control groups based on previous research

##### Evidence-based intervention provided to patients

Assuming a design effect due to clustering of 2 based on previous studies [30-33], a sample of 188 patients in each group will detect a 20% difference in the proportion of patients offered evidence-based interventions (motivational counselling or referral to appropriate services) to modify their risk factors, compared to the control group.

##### Reduction in physiological risk factors

Assuming a design effect due to clustering of 1.8, loss to follow up of 20% and standard deviations based on data from general practice diabetes registers [34]:

• weight: assuming a standard deviation of 19, a sample size of 382 will be sufficient to detect a mean difference of 4 Kg in weight between intervention and control groups.

• LDL cholesterol: assuming a standard deviation of 0.9, a sample size of 342 will be sufficient to detect a mean difference of 0.2 mmol/L in LDL-cholesterol between intervention and control groups.

• blood pressure: assuming a standard deviation of 11, a sample size of 228 will be sufficient to detect a mean difference of 3 mmHg in systolic blood pressure between intervention and control groups.

##### Reduction in behavioural risk factors

Assuming a design effect due to clustering of 1.8 and a 20% loss to follow up with estimates based on previous research [35]:

• if patients eat a standard deviation 2.1 portions of fruit and vegetables a sample of 288 in each group would have sufficient power to detect a mean 0.5 portion difference.

• if 22% smoke, a sample of 363 in each group would have sufficient power to detect a 6% difference.

• if 30% consume alcohol at risk levels, a sample of 362 in each group would have sufficient power to detect a 7% difference.

• For physical activity, a score of 2.1 in a sample of 305 in each group would have sufficient power to detect a mean 0.5 score difference.

**Figure 3:**
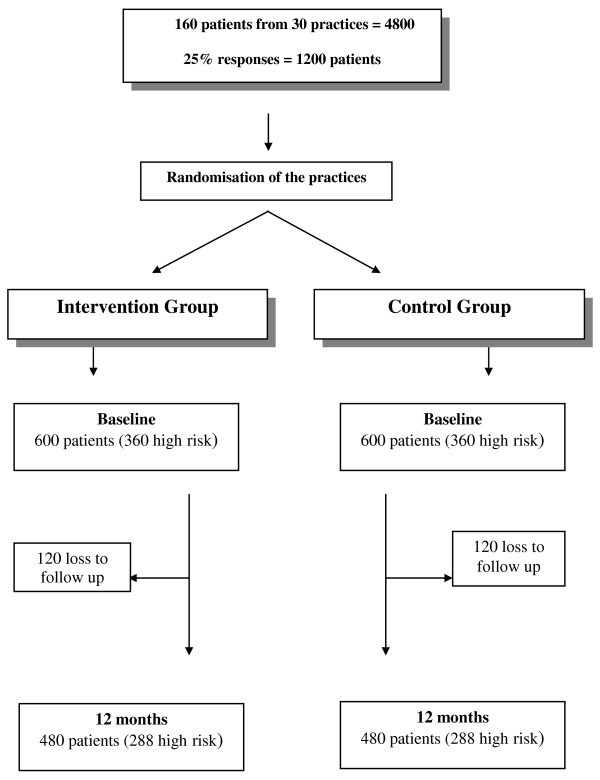
**Predicted patient recruitment**.

We aim to recruit 30 general practices and identify 160 patients from each practice (total = 4800). From this we anticipate 25% responses in each group (600 patients in each group). An estimated 20% loss to follow-up will leave 480 patients in each group, of which we estimate 60% are high risk (288 patients in each group) (Figure 3).

### Data Analysis

#### Quantitative evaluation - statistical analysis

Quantitative data analysis will be conducted using SPSS and ML-Win (Multilevel statistics for Windows). Multilevel linear models will be used for continuous response variables and multilevel logistic models for binary responses. Co-variates will be included in the analyses to adjust for baseline differences between the intervention and control groups.

The analysis will include changes in the study variables before and after the intervention in both intervention and control groups, including changes in patients' lifestyle risk factors and comparison of outcomes at 12 months between the intervention and control groups after adjusting for baseline differences.

#### Qualitative evaluation

As this is a complex intervention delivered within the context of normal practice, a qualitative evaluation of the implementation process is important in interpreting the study findings [36]. Semi-structured interviews will be conducted with intervention GPs, PNs and IOs to explore their views on (a) the *CHANGE for HIPS *intervention program, (b) experiences of implementation (including feasibility, barriers and facilitators), (c) perceptions of patients' views/experiences of participation and (d) key lessons for dissemination of the program into routine service provision. All interviews will be transcribed verbatim and subject to thematic analysis using NVivo 8 [37] to identify convergence and divergence of themes.

### Pilot Study

All procedures and tools used in the study have been pilot tested with a practice in a nonparticipating DGP.

### Ethics

The project has been approved by the University of New South Wales Human Research Ethics Committee (HREC).

## Discussion

Much of the focus in general practice primary preventive care has been on assessment of risk, use of medications, and brief interventions to address single risk factors. While trials have demonstrated the effectiveness of intense interventions to prevent diabetes or cardiovascular disease, it has been difficult to replicate this within routine practice. This is one of the first studies in Australia to evaluate an intervention which includes assessment and brief intervention in routine general practice coupled with a more intensive referral-based intervention based in the local DGP. The trial is unique in that the intervention is delivered as part of routine practice (both in general practices and the DGP), and the study aims to examine its impact in both high and low risk patients. The study will also provide valuable qualitative data on the barriers and facilitators to implementing brief intervention and referral of high risk patients to more intensive intervention in the general practice setting.

The study involved a strong collaborative partnership with DGPs and non-government organizations such as the National Heart Foundation. These relationships are essential not only to recruit participants into the study but also to deliver an effective intervention and subsequently implement the findings.

The results of this study will help inform improvements to the implementation of primary prevention of vascular disease and inform the implementation of other preventive health initiatives which involve referral to group programs outside general practice, such as the 45-year-old health check and the diabetes risk assessment in Australian general practice. The findings of the study will be disseminated using peer-reviewed journals, conference presentations, and research summaries tailored for practitioners, service managers and policy makers. We anticipate that it will inform broader policies and strategies including:

• defining the optimal roles of general practice staff, allied health providers and group referral programs in providing interventions for low and high risk patients

• redesigning the pathways for referral and follow up of high risk patients

• providing more effective support for primary prevention through DGPs in general practice;

• redesigning the Medicare Benefits Schedule (MBS) preventive care related item numbers;

• integrating the complex preventive interventions required to prevent chronic diseases such as diabetes within routine general practice and its associated services and programs.

## Competing interests

The authors declare that they have no competing interests.

## Authors' contributions

All authors have contributed to study design and have reviewed and approved the final manuscript. In particular, MFH (CI) has developed research protocol, MFH, MP, DL, and MF leadership and oversight of research, MF and MFH have led the development of data collection tools and processes, RAL and MFH and MF have developed intervention design.

## Acknowledgements

The authors would like to acknowledge the National Health and Medical Research Council (NHMRC) for funding the study. All authors are funded by Universities. This paper is presented on behalf of the HIPS team which include: E.Rix, H. Schutze; J.Clark; K. Foran; K. Revelas, E.Saurman; M.Furneaux; and other investigators in HIPS which include G.Heading, C.Tzarimas, N. Zwar, and U. Jayasinge.

## Pre-publication history

The pre-publication history for this paper can be accessed here:

http://www.biomedcentral.com/1471-2296/11/57/prepub
